# Organacidia Gastrica

**Published:** 1902-09-27

**Authors:** 


### ORGANACIDIA GASTRICA.

Dr. Mark I. Knapp,[1] of New York, describes a disease which he calls organacidia gastrica. Its non-discovery has been due to the difficulty which exists in distinguishing between the organic acids and HCl, leading to these cases of hyperacidity being lumped together as cases of hyperchlorhydria. His observations, however, go to suggest that real hyperchlorhydria is not of such frequent occurrence as some have thought. Normally there should not be any, or possibly only a trace of organic acids in the stomach, and with the normal quantity of free HCl a small quantity of such acids may cause no inconvenience. But when their quantity increases, or the free HCl diminishes in quantity, distressing symptoms are produced. Dr. Knapp divides the cases under consideration into three groups:—(1) Simple cases in which the organic acids have been introduced in food, as in fruit, pickles, salads, strong cheese, etc. As a rule in such cases the symptoms disappear after the use of a purgative or an emetic; (2) cases in which moulds are present in the stomach; these he speaks of as cases of gastrosia fungosa, and (3) cases in which the yeast plant develops in the stomach—zymosis gastrica. Gastrosia fungosa is the most common of the three forms. In the diagnosis of this condition he urges the careful use of the microscope and a very complete chemical examination of the matters returned after a test meal. The symptoms are largely those of acid dyspepsia. What appear to be the important points made by Dr. Knapp are his insistence on the injurious effects of the presence of organic acids in the stomach, and on the fact that the origin and continuance of the disease is largely due to the habit of eating more or less mouldy food. He says " no food is exempt from mouldiness." Food should be eaten soon after it is cooked. "No family should cook more than is required for the next meal, and should destroy what is left over. The poor especially should be warned not to eat any food left over from the day before." Sweets, stewed fruit, jams, and pies should not be allowed, and finally, all meat should be thoroughly cleaned and washed before cooking. Dr. Knapp strongly recommends that, except where meat is to be broiled, it should be dealt with according to the Jewish custom of making it "kosher." This consists, he says, in first soaking the meat in plenty of clean water for about twenty minutes, and then salting it thoroughly. After the salting it is put on a seive-like tray so as to allow free drainage, and in about thirty minutes it is again thoroughly washed to remove the excess of salt. Then only is it ready for boiling, stewing, etc.

The mechanical treatment of the disease is best carried out by lavage, while in the medicinal treatment the important thing is not to fly to carbonate of soda, which, by combining with the HCl, only makes matters worse, but to give mineral acids by which the organic acids are decomposed. There is a good deal to be said for the practice of neither eating nor drinking anything but what has been quite recently cooked—or, in other words, sterilised—and Dr. Knapp does but put in a forcible manner a line of thought which is taking form on many sides in regard to the injury which may result from trusting too much to the bactericidal powers of the gastric and other secretions.

[1] Med. Rec., Sept. 6.

